# Targeting the Gut in Sepsis: Therapeutic Potential of Medical Gases

**DOI:** 10.3390/biom16020199

**Published:** 2026-01-28

**Authors:** Tetsuya Yumoto, Takafumi Obara, Hiromichi Naito, Atsunori Nakao

**Affiliations:** Department of Emergency, Critical Care, and Disaster Medicine, Faculty of Medicine, Dentistry, and Pharmaceutical Sciences, Okayama University, 2-5-1 Shikata-cho, Kita-ku, Okayama 700-8558, Japan; dainosinn@gmail.com (T.O.); naito05084@gmail.com (H.N.); qq-nakao@okayama-u.ac.jp (A.N.)

**Keywords:** carbon monoxide, gastrointestinal tract, gut, hydrogen, hydrogen sulfide, sepsis, septic shock

## Abstract

Sepsis is a life-threatening condition characterized by a dysregulated host response to infection, often resulting in multiorgan dysfunction. Among affected systems, the gastrointestinal tract plays a central role in sepsis progression by promoting systemic inflammation through impaired barrier function, immune imbalance, and microbiome alterations. Recent research has identified selected medical gases and gasotransmitters as promising therapeutic candidates for preserving gut integrity in sepsis. In particular, hydrogen, carbon monoxide, and hydrogen sulfide exhibit antioxidative, anti-inflammatory, and cytoprotective properties. These gases act through defined molecular pathways, including activation of Nrf2, inhibition of NF-κB, and preservation of tight junction integrity, thereby supporting intestinal barrier function. In addition, they influence immune cell phenotypes and autophagy, with indirect effects on the gut microbiome. Although most supporting evidence derives from preclinical models, translational findings and emerging safety data highlight the potential of gut-targeted gas-based strategies. This review summarizes current mechanistic and translational evidence for gut-protective medical gases in sepsis and discusses their integration into future organ-specific and mechanism-based therapeutic approaches.

## 1. Introduction

Sepsis remains a major global health concern, with an estimated 49 million incident cases and 11 million deaths in 2017, accounting for nearly 20% of all global deaths [1]. It is defined as a life-threatening condition characterized by organ dysfunction resulting from a dysregulated host response to infection [2]. Despite advances in supportive care, targeted therapies for sepsis remain elusive [3]. One of the major challenges in developing targeted therapies is the marked heterogeneity among patients, including differences in host factors, infectious sources, causative microbes, and clinical trajectories [4,5,6]. Nevertheless, sepsis is fundamentally an inflammatory disorder driven by the activation of the innate immune system [7,8,9].

Increasing attention has turned to the gastrointestinal tract as a central player in sepsis pathogenesis. The gut epithelium, microbiota, and mucosal immune system interact to maintain barrier integrity and immune tolerance. Sepsis-induced intestinal hyperpermeability and dysbiosis contribute to microbial translocation and systemic inflammation, reinforcing the concept of the gut as a “motor” of sepsis [10,11,12,13,14,15,16].

Given the central role of dysregulated inflammation in sepsis, gaseous agents have emerged as promising therapeutic candidates [17,18,19,20]. However, the term “medical gases” has been used inconsistently [21,22]. Here, we distinguish medical gases from gasotransmitters as distinct but partially overlapping categories based on regulatory status and endogenous origin. In this context, nitric oxide (NO), carbon monoxide (CO), and hydrogen sulfide (H_2_S) are regarded as the core, universally accepted gasotransmitters [23]. Although NO plays critical roles in sepsis pathophysiology, its direct gut barrier-protective effects in sepsis are less well defined; therefore, the present review focuses primarily on CO and H_2_S. In addition, molecular hydrogen (H_2_), which is administered therapeutically as a medical gas rather than a gasotransmitter, has attracted particular interest and is also discussed in detail in this review.

Beyond their traditional roles, these gases act as signaling molecules with anti-inflammatory, antioxidative, and cytoprotective properties. Low-dose CO and H_2_ have shown potential to suppress proinflammatory cytokines and reduce organ injury in preclinical sepsis models [24,25]. H_2_S also exhibit organ-protective effects through modulation of inflammatory and cellular stress pathways [26]. Although still largely investigational, these gases represent a novel, mechanism-based approach to mitigating sepsis-induced organ dysfunction.

Notably, the therapeutic potential of medical gases has begun to progress from bench to bedside. H_2_ has shown a favorable safety profile across multiple preclinical models and various clinical conditions, prompting calls for well-powered trials in critical illness, although no randomized studies have yet evaluated H_2_ in sepsis [27]. CO in mechanically ventilated patients with sepsis-induced acute respiratory distress syndrome demonstrated that precise administration was feasible and well tolerated, without administration-related adverse events or carboxyhemoglobin levels exceeding 10% [28]. H_2_S therapies remain largely preclinical, but translation is supported by clinically used donors such as sodium thiosulfate, which releases H_2_S in vivo and is being studied in several ongoing trials [29]. Together, these developments highlight the promise of medical gases while underscoring the need for rigorous clinical studies to define dosing, delivery, and patient selection in sepsis.

Accordingly, this review focuses on the role of selected medical gases and gasotransmitters in protecting gut integrity during sepsis, highlighting mechanisms including tight junction (TJ) regulation, autophagy, immune modulation, and microbiome preservation.

## 2. Gut Dysfunction in Sepsis

Sepsis-induced gut injury encompasses multiple, interlinked processes. For clarity, this section is organized into three subthemes: barrier disruption, immune dysregulation and microbiome alterations.

### 2.1. Mechanisms of Barrier Disruption

Of note, the gastrointestinal tract is central to sepsis pathogenesis, often termed the “motor” of systemic inflammation [30]. Disruption of the intestinal barrier permits translocation of microbes and endotoxins, triggering systemic inflammation and multi-organ dysfunction. Gut barrier dysfunction in sepsis is characterized by increased paracellular permeability due to the disassembly of TJs, including key proteins such as claudins, zonula occludens-1 (ZO-1), and occludin [14,15,31,32]. Sepsis-induced TJ disruption is further exacerbated by the phosphorylation of cytoskeletal proteins like myosin light chain (MLC), contributing to epithelial barrier collapse [33,34].

### 2.2. Immune Dysregulation

Sepsis involves a complex interplay of hyperinflammation and immunosuppression [35,36]. Initially, pattern recognition receptors activate inflammatory cascades, leading to cytokine release, endothelial dysfunction, and vascular leakage, causing tissue edema and organ injury. Neutrophils and platelets exacerbate damage via neutrophil extra-cellular traps formation and immunothrombosis [37]. As sepsis progresses, immune suppression emerges, marked by lymphocyte apoptosis, monocyte reprogramming, and upregulation of checkpoint receptors (e.g., programmed cell death protein-1, cytotoxic T-lymphocyte associated protein 4) [38,39,40]. This dual-phase dysregulation impairs pathogen clearance and increases susceptibility to secondary infections, contributing to prolonged disease and chronic critical illness [7,35].

### 2.3. Microbiome Alterations

Moreover, the gut microbiome plays a critical role in sepsis pathophysiology, influencing immune regulation, infection susceptibility, and organ dysfunction. Antibiotic treatment during sepsis often disrupts microbial homeostasis, leading to loss of commensals, expansion of pathogenic bacteria, and reduced short-chain fatty acids, thereby worsening outcomes [41]. In parallel, gut mycobiome dysbiosis has been observed in critically ill patients with sepsis or trauma. This includes an overgrowth of Candida species and a depletion of beneficial fungi such as Saccharomyces, which may persist for weeks and contribute to a pathobiome state that promotes infection [42]. Both bacterial and fungal disruptions are linked to immune suppression, metabolic shifts, and increased risk of secondary infections and chronic critical illness. Current evidence supports interventions such as antibiotic stewardship, fiber-enriched nutrition, and emerging therapies like fecal microbiota transplantation, though these remain largely investigational [43].

### 2.4. Clinical Heterogeneity and Therapeutic Implications

Clinically, sepsis is highly heterogeneous, with phenotypes such as inflammopathic, coagulopathic, and adaptive subtypes linked to different outcomes and treatment responses [44,45,46]. Despite these insights, no single biomarker reliably guides diagnosis or therapy [4,47]. Disruption of the gut microbiota further impairs barrier integrity and immune balance, reinforcing the “gut-origin of sepsis” hypothesis [16,48]. Taken together, these findings highlight the gastrointestinal tract as both a driver and target of systemic inflammation. Therapeutic strategies that restore gut barrier function, immune balance, and microbiota homeostasis are urgently needed. Medical gases, with their multifaceted effects on epithelial and immune cell function, offer a compelling avenue for gut-targeted interventions in sepsis.

## 3. Overview of Medical Gases

Medical gases represent a diverse group of biologically active molecules with growing interest as therapeutic agents in sepsis [17]. These gases include not only traditional respiratory gases but also endogenous gasotransmitters and noble gases that exhibit unique physicochemical and biological properties, although the terminology has been used inconsistently in the literature [21,22]. Their small molecular size and high diffusibility enable rapid tissue penetration and intracellular signaling, offering advantages in critical illness characterized by inflammation, oxidative stress, ischemic–reperfusion injury, and organ dysfunction [21,49,50]. For clarity, in this review, medical gases and gasotransmitters are considered conceptually distinct but partially overlapping categories, and several representative gases currently under investigation or in clinical practice are outlined below.

In addition to H_2_, CO, and H_2_S, clinically established medical gases such as NO, nitrous oxide, and nitrogen are widely used in critical care. However, while NO plays important roles in vascular and immune regulation, and nitrous oxide and nitrogen are primarily employed for anesthetic or ventilatory purposes, direct evidence supporting gut-targeted protective signaling by these gases in sepsis is limited. Accordingly, they are not discussed in detail in the present review.

### 3.1. CO

CO is generated endogenously via heme degradation by heme oxygenase (HO) isoforms HO-1 (inducible) and HO-2 (constitutive) [51]. In this physiologic context, CO acts as a signaling molecule that modulates mitochondrial function, apoptosis and inflammation. While CO is toxic at high concentrations due to its strong binding to hemoglobin, low levels act as a signaling molecule with anti-inflammatory, anti-apoptotic, and vasoregulatory effects. Its therapeutic potential lies in modulating oxidative stress and preserving cellular homeostasis. For clinical use, CO can be delivered via precisely controlled, low-dose inhalation or through carbon monoxide-releasing molecules (CORMs), which are engineered to release CO in a targeted manner while minimizing carboxyhemoglobin formation. Examples of these agents include metal-based CORM-2 and CORM-3 and the manganese-based CORM-401 for intracellular CO release [52,53]. These agents are used in preclinical models of sepsis, ischemia–reperfusion injury, and inflammation to harness the therapeutic effects of CO while minimizing toxicity, with ongoing efforts to improve their delivery and safety for clinical use.

### 3.2. H_2_

H_2_ is an inert, non-polar molecule that is produced in trace amounts in the human body, particularly by intestinal flora [54]. Importantly, H_2_ is not classified as a gasotransmitter, as mammalian cells lack enzymatic machinery for its endogenous production, and is instead discussed here as a therapeutically administered medical gas. Its most notable characteristic is its selective antioxidant activity, especially against hydroxyl radicals and peroxynitrite [49]. Unlike many antioxidants, H_2_ does not interfere with physiological signaling reactive oxygen species (ROS). Its high diffusibility allows it to reach intracellular targets such as mitochondria and nuclei with ease. In ischemia–reperfusion models, including crush syndrome or transplantation, H_2_ protects tissues by reducing oxidative stress, preserving mitochondrial function, suppressing proinflammatory cytokines, and activating cytoprotective pathways such as nuclear factor erythroid 2-related factor 2 (Nrf2)/HO-1 and B-cell lymphoma 2 [55,56,57].

Endogenously, trace amounts of H_2_ are generated in the gastrointestinal tract through fermentation by H_2_-producing gut microbiota; hydrogenases convert carbohydrates to H_2_ and short-chain fatty acids. For therapeutic purposes, H_2_ can be administered exogenously via low-concentration inhalation, consumption of H_2_-rich water, injection of H_2_-rich saline. Endogenous production can also be enhanced by prebiotic substrates such as inulin or lactulose that feed H_2_-producing bacteria [58].

### 3.3. H_2_S

Endogenous H_2_S synthesis occurs via three key enzymes: cystathionine-β-synthase Endogenous H_2_S synthesis occurs via three key enzymes: cystathionine-β-synthase (CBS), cystathionine-γ-lyase (CSE), and 3-mercaptopyruvate sulfurtransferase, which convert L-cysteine and homocysteine to H_2_S in the cytosol and mitochondria [26]. Although gaseous H_2_S is toxic at high levels, pharmacologic delivery through donors such as sodium thiosulfate offers a safer therapeutic strategy, particularly under hypoxic conditions where H_2_S production is enhanced [59,60]. Exogenous delivery commonly employs sulfide salts (NaHS, Na_2_S) or slow-releasing donors such as GYY4137 and AP39, which allow more controlled H_2_S release. Sodium thiosulfate (Na_2_S_2_O_3_) also functions as a clinically used H_2_S donor with minimal side effects and is under investigation as a safer therapeutic source of H_2_S in critical illness [61]. These therapeutic approaches differ fundamentally from the tightly regulated endogenous production of H_2_S.

Together, these gases represent a novel therapeutic frontier in sepsis. Their diverse modes of action, which include antioxidant and anti-inflammatory effects as well as mechanical support of ventilation, underscore their potential to modulate critical pathways involved in sepsis pathophysiology. To explore how medical gases may protect gut integrity in sepsis, this review first outlines their effects on key components of the intestinal barrier. Among various medical gases, H_2_, CO, and H_2_S have demonstrated the most consistent and promising gut-protective effects across multiple preclinical studies. Therefore, while an overview of several gases has been provided, the subsequent sections focus primarily on these three agents. Their actions are discussed in terms of epithelial barrier preservation, immune modulation, autophagy regulation, and microbiome homeostasis [16,62].

### 3.4. Methane

Methane (CH_4_) has recently attracted attention as an emerging candidate gaseous mediator [63]. In addition to its well-established production by anaerobic gastrointestinal archaea, experimental evidence suggests that CH_4_may also be generated through non-enzymatic reactions associated with oxidative stress in mammalian systems. Preclinical studies have reported anti-inflammatory and mitochondrial-protective effects of exogenously administered CH_4_ in sepsis models, including suppression of NF-κB signaling and preservation of epithelial barrier function [64]. However, CH_4_ is not yet universally accepted as a core gasotransmitter, and human data remain limited. Accordingly, CH_4_ is discussed here to provide conceptual context rather than as a primary focus of the present review.

From a regulatory perspective, medical gases such as oxygen, medical air, nitrogen, nitrous oxide, carbon dioxide, and helium are defined as medicinal products administered for therapeutic, anesthetic, or diagnostic purposes in controlled clinical settings [65]. Among these, oxygen is indispensable in the management of critically ill patients with sepsis; however, its effects on gastrointestinal physiology are complex and context dependent.

The intestinal lumen is physiologically hypoxic, and disruption of this low-oxygen environment may adversely affect epithelial metabolism and microbial ecology. Emerging evidence suggests that liberal systemic oxygen therapy aimed at high arterial oxygen saturation can exacerbate intestinal barrier dysfunction, promote microbiome dysbiosis, and amplify inflammatory signaling cascades [66]. Increased luminal oxygen tension may favor the expansion of oxygen-tolerant pathogenic bacteria, thereby aggravating gut injury and systemic inflammation [67].

In contrast, experimental studies have demonstrated that targeted oxygen delivery to the intestinal lumen can mitigate non-occlusive ischemia-induced gut injury and sepsis-related organ dysfunction without inducing systemic hyperoxia [68]. In addition, medical air, a defined mixture of nitrogen and oxygen, has been proposed as a tool for conservative oxygen therapy, with the aim of maintaining adequate tissue oxygenation while minimizing oxidative and microbiome-related adverse effects [69].

Together, these findings highlight that classical medical gases, particularly oxygen and medical air, are highly relevant to gut-related pathophysiology in sepsis. While these gases are not the primary focus of the present mechanistic review, their inclusion provides essential clinical and regulatory context for gut-targeted gas-based strategies.

An overview of the mechanistic, preclinical, and clinical evidence supporting gut-targeted medical gases is summarized in Table 1.

## 4. Effects of Medical Gases on TJs and Epithelial Barrier Integrity

Disruption of TJs is a hallmark of sepsis-induced intestinal barrier dysfunction, contributing to microbial translocation and systemic inflammation. Emerging evidence suggests that medical gases, particularly H_2_, CO, and H_2_S, help preserve epithelial integrity by modulating oxidative stress, controlling inflammation, and stabilizing cytoskeletal structure [10,70].

### 4.1. H_2_

H_2_ demonstrates potent protective effects on the intestinal epithelium by stabilizing TJ proteins and reducing permeability. In murine sepsis models, inhaled H_2_ preserves occludin and ZO-1 expression and suppresses epithelial apoptosis and oxidative injury. These effects are largely mediated through activation of the Nrf2/HO-1 pathway, which attenuates the release of high mobility group box 1 (HMGB1), a late proinflammatory mediator implicated in TJ disruption. In Nrf2-deficient mice, H_2_ fails to confer protection, confirming the importance of this signaling axis [70,71,79]. H_2_ fails to confer protection, confirming the importance of this signaling axis [62,63,64]. H_2_-rich saline has also shown efficacy, improving survival, reducing bacterial translocation, and maintaining TJ integrity while downregulating tumor necrosis factor-alpha (TNF-α), interleukin (IL)-1β, IL-6, inducible nitric oxide synthase, and lipid peroxidation markers [72]. H_2_ further limits the overgrowth of Enterobacteriaceae and dampens nuclear factor Kappa B (NF-κB) and mitogen-activated protein kinase (MAPK) activity, thereby reinforcing gut barrier function. Unlike gasotransmitters, H_2_ acts as a diffusible antioxidant, selectively scavenging cytotoxic radicals like hydroxyl and peroxynitrite without interfering with physiological ROS signaling. Both inhaled and luminal delivery routes have demonstrated effectiveness in preserving intestinal integrity during sepsis.

### 4.2. CO

CO, primarily produced endogenously via HO-1, exhibits protective effects on the intestinal barrier that are well characterized in sepsis models. CO (administered by CO-releasing molecules like CORM-2 or via HO-1 induction) preserves TJ structure and function by modulating key inflammatory pathways. In a rat cecal ligation and puncture (CLP) model, CORM-2 prevented sepsis-induced TJ protein loss (maintaining levels of ZO-1, occludin and claudin-1) and ultrastructural TJ damage. CO therapy also normalized F-actin–myosin dynamics: septic controls showed elevated MLC phosphorylation and actin contraction, whereas CO-treated rats had significantly lower phosphorylated-MLC levels and preserved junctional morphology [74]. The mechanisms center on CO’s anti-NF-κB and anti-MAPK activities. CO/HO-1 signaling inhibits the degradation of IκB-α and nuclear translocation of NF-κB p65, thereby reducing MLC kinase (MLCK) transcription and subsequent MLC-2 phosphorylation [75]. In parallel, HO-1-derived CO can attenuate p38 and c-Jun N-terminal kinase (JNK)-MAPK pathways (often activated by sepsis toxins), which further contributes to stabilizing TJs [76]. The HO-1/CO axis thus “locks” the intestinal barrier by both upregulating TJ proteins and preventing their disassembly. While therapeutic CO delivery in humans is complex due to toxicity, these findings underscore a clear role for CO in reinforcing epithelial barrier integrity under septic stress.

### 4.3. H_2_S

Endogenously produced by enzymes such as CBS and CSE, H_2_S has shown gut-protective properties in sepsis [80]. Through persulfidation of regulatory proteins such as Keap1, H_2_S activates Nrf2 signaling and enhances antioxidant responses. Slow-releasing H_2_S donors like GYY4137 also suppress NF-κB activity and inflammasome assembly, thereby decreasing proinflammatory cytokines. A distinct feature of H_2_S is its inhibition of the MLCK-MLC phosphorylation pathway, which prevents TJ opening and actomyosin ring contraction [17]. In both sepsis and chronic injury models, H_2_S preserves occludin, claudins, and ZO-1 expression and maintains epithelial barrier function. While high concentrations of H_2_S can be toxic, low-dose formulations harness its cytoprotective potential effectively.

**Figure 1:**
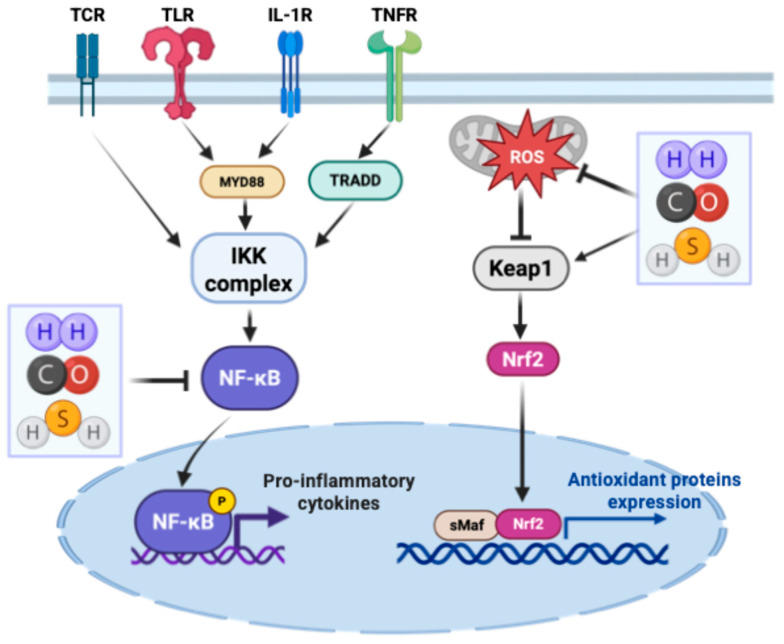
Anti-inflammatory and anti-oxidative pathways targeted by medical gases in sepsis.

**Figure 2:**
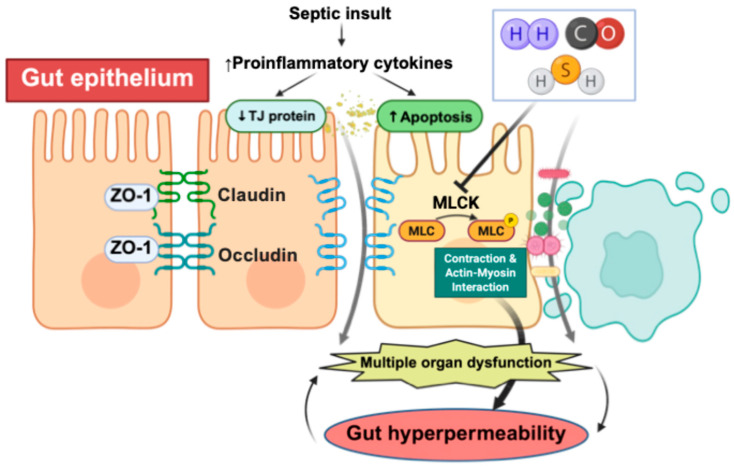
Stabilization of TJs and suppression of MLCK-mediated barrier disruption by medical gases.

The anti-inflammatory, anti-oxidative, and MLCK-dependent TJ-stabilizing effects of these gases are illustrated in Figure 1 and Figure 2.

This schematic illustrates how hydrogen (H_2_), carbon monoxide (CO), and hydrogen sulfide (H_2_S) attenuate inflammatory signaling and promote antioxidant responses during sepsis. Medical gases inhibit the activation of the IKK–NF-κB pathway triggered by TCR, TLR, IL-1R, and TNFR signaling, thereby reducing downstream pro-inflammatory gene expression. In parallel, the gases suppress ROS-driven Keap1 activation and enhance Nrf2-dependent transcription of antioxidant proteins. Together, these effects dampen inflammatory cytokine production and strengthen cellular defense against oxidative stress.

Septic insult increases pro-inflammatory cytokines and apoptosis, leading to reduced expression of TJ proteins (ZO-1, occludin, claudins) and activation of MLCK, which drives actin–myosin contraction and barrier failure. Hydrogen (H_2_), carbon monoxide (CO), and hydrogen sulfide (H_2_S) counteract these processes by preserving TJ architecture, inhibiting MLCK activation, and mitigating epithelial injury. These combined actions limit gut hyperpermeability and protect against downstream multiple organ dysfunction.

### 4.4. Comparative Mechanistic Perspective

Despite their distinct modes of action, such as H_2_ acting as a diffusible antioxidant, H_2_S exerting effects through protein persulfidation, and carbon monoxide operating via HO-1 signaling, all three gases converge on the preservation of TJ structure by enhancing antioxidant defenses, suppressing inflammatory signaling (e.g., NF-κB), and reducing cytoskeletal tension. Their shared capacity to stabilize key TJ proteins (occludin, claudins, ZO-1) and inhibit junctional disassembly underscores their therapeutic potential for gut-targeted strategies in sepsis. These overlapping yet complementary mechanisms suggest opportunities for tailored or combination approaches in future clinical applications.

In addition to their shared ability to suppress NF-κB activation and enhance antioxidant defenses, each medical gas engages distinct upstream mechanisms that shape its biological effects. H_2_ promotes autophagy and mitophagy, thereby attenuating nucleotide-binding domain, leucine-rich–containing family, pyrin domain-containing-3 (NLRP3) inflammasome activation and pyroptosis in sepsis models, a pathway that is independent of its classical antioxidant properties and distinguishes it from the other gases [81]. CO, by contrast, uniquely activates soluble guanylate cyclase and calcium-activated potassium channels, and further exerts protective effects through Beclin-1-dependent autophagy and enhanced phagocytic clearance of bacteria, highlighting mechanisms not shared by H_2_ or H_2_S [77,82]. H_2_S operates through yet another pathway, exerting its redox-regulatory influence via protein persulfidation; in particular, H_2_S modifies Keap1 at Cys151, promoting dissociation of the Keap1-Nrf2 complex and driving antioxidant gene activation through a mechanism unique to this gasotransmitter [83]. Together, these distinct yet complementary mechanisms illustrate that H_2_, CO, and H_2_S converge on similar downstream protective effects through fundamentally different molecular entry points.

## 5. Modulation of Gut Immune Responses via Medical Gases

Dysregulation of the gut’s immune homeostasis is a critical aspect of sepsis pathophysiology. An initial hyper-inflammatory response in the intestinal mucosa can drive excessive cytokine release and tissue injury, while subsequent immunosuppression increases vulnerability to secondary infections. Emerging evidence suggests that medical gases including H_2_, H_2_S, and CO can help rebalance gut immune responses by tempering excessive inflammation and promoting resolution, all while bolstering the host’s ability to clear pathogens. These gases modulate key signaling pathways (e.g., NF-κB, MAPKs, and Nrf2) and cytokine profiles in the gut, thereby preventing immune overactivation and maintaining mucosal immune equilibrium during septic challenge.

### 5.1. H_2_

H_2_ gas exhibits broad anti-inflammatory effects in the gut during sepsis. In animal sepsis models, H_2_ therapy significantly lowers pro-inflammatory cytokine levels (e.g., TNF-α, IL-6) and late mediators like HMGB1, while upregulating the anti-inflammatory cytokine IL-10 [84]. These shifts are accompanied by a phenotypic modulation of immune cells: H_2_ exposure tends to reduce pro-inflammatory M1 macrophage polarization and promote the emergence of anti-inflammatory M2 macrophages. Such immunomodulation is reflected in improved outcomes. For instance, septic mice receiving 2% H_2_ show higher survival rates and less intestinal damage from oxidative stress and inflammation [85]. Mechanistically, H_2_’s benefits critically depend on the Nrf2/HO-1 pathway: H_2_ inhalation induces HO-1 in intestinal tissues and concomitantly suppresses HMGB1 release, an effect that is abrogated in Nrf2-knockout mice. By activating Nrf2, H_2_ boosts antioxidant defenses and limits downstream inflammatory signaling. Additionally, H_2_ directly scavenges the most cytotoxic reactive oxygen species (such as hydroxyl radicals and peroxynitrite), curbing oxidative activation of immune cells without impeding physiologic ROS signals. This unique action (distinguishing H_2_ from canonical “gasotransmitters” like CO and H_2_S) leads to attenuation of NF-κB and MAPK pathway activity, resulting in reduced transcription of inflammatory genes and protection of the gut from cytokine-mediated injury [86]. Together, these effects position H_2_ as a diffusible immunomodulator that selectively dampens harmful inflammation while preserving or even enhancing essential host defenses in the gut.

### 5.2. CO

CO exerts multifaceted immunomodulatory effects in the gut during sepsis. Delivered exogenously through CO-releasing molecules or induced endogenously by HO-1, CO supports intestinal immune homeostasis by rebalancing cytokine expression. It suppresses TNF-α, IL-1β, IL-6, and IL-17, while enhancing IL-10 and IL-22 [87]. This cytokine shift is mediated by CO’s inhibition of NF-κB activation (through stabilization of IκB-α and reduced p65 translocation) and suppression of stress kinases such as p38 and JNK, ultimately lowering the transcription of pro-inflammatory genes. CO also reinforces gut barrier integrity by fostering an anti-inflammatory environment, with IL-10 acting both as an effector and inducer in a protective feedback loop. Beyond its anti-inflammatory role, CO enhances host defense by improving macrophage bactericidal activity through increased phagolysosomal acidification, which in turn reduces bacterial translocation to mesenteric lymph nodes and systemic organs [88]. These immunoregulatory actions involve diverse cell types, including macrophages, dendritic cells, T cells, and epithelial cells, highlighting the HO-1/CO axis as a promising therapeutic target for restoring immune equilibrium in the septic gut.

### 5.3. H_2_S

H_2_S is recognized as an important immune regulator in the gut. Exogenous H_2_S therapy in sepsis has demonstrated striking protective effects: it increases survival and ameliorates systemic inflammation in septic mice, in part by limiting immune cell apoptosis (e.g., in the spleen) and accelerating bacterial clearance [80]. H_2_S exerts a potent anti-inflammatory influence on gut immune responses through multiple mechanisms. It suppresses the NF-κB signaling cascade, as H_2_S donors inhibit IκBα degradation and p65 nuclear translocation, thereby reducing the transcription of pro-inflammatory genes [89]. At the same time, H_2_S can prevent over-activation of the NLRP3 inflammasome complex: for example, the slow-releasing H_2_S donor GYY4137 was shown to reduce expression of NLRP3 inflammasome components (apoptosis-associated speck-like protein containing a CARD, caspase-1) in septic tissues, correlating with lower IL-1β levels [90]. In a murine sepsis model, GYY4137 also markedly decreased neutrophil infiltration into organs such as the lungs and mitigated tissue damage, confirming the ability of H_2_S to restrain excessive leukocyte-driven inflammation. Dosage and context are critical, however: excessive or rapid-release H_2_S can have a pro-inflammatory effect (endogenous H_2_S surges in sepsis were shown to drive NF-κB activation and cytokine overproduction) [91]. In summary, H_2_S serves as a double-edged sword for gut immunity, when properly modulated, it reinforces antioxidant defenses, quells unwarranted inflammation (including inflammasome-driven pathways), and supports the host’s antimicrobial capacity.

## 6. Medical Gases and Gut Microbiome Regulation

The gut microbiome plays a critical role in regulating host immunity, maintaining epithelial barrier integrity, and protecting against pathogenic invasion [92,93]. In the setting of sepsis, microbial diversity and functional capacity are often disrupted, leading to pathobiont overgrowth, reduced production of beneficial metabolites such as short-chain fatty acids, and heightened inflammatory responses. These alterations contribute to barrier dysfunction, systemic inflammation, and multiorgan failure. Consequently, preserving or restoring microbiome homeostasis has emerged as a potential therapeutic strategy, positioning the gut as both a contributor to and a target of sepsis pathophysiology.

Among microbiota-related factors, H_2_-producing gut bacteria have gained attention for their overlooked health benefits [94]. These microbes not only scavenge oxidative stress but also support epithelial barrier integrity and modulate host immunity. Their activity has been associated with protection against inflammatory bowel disease and neurodegeneration, possibly via gut–brain axis signaling and longevity promotion. H_2_ therapy, through inhalation or H_2_-rich saline, has demonstrated protective effects on the gut microbiome in septic models. Ikeda et al. showed that H_2_-rich saline improved survival by suppressing *Enterobacteriaceae* overgrowth, reducing bacterial translocation, and mitigating intestinal hyperpermeability and inflammation [72]. Inhaled H_2_ also increased the abundance of beneficial bacteria such as *Akkermansia* and enhanced microbial diversity, while modulating fecal metabolite profiles toward neuroprotective and anti-inflammatory states [95]. These findings suggest H_2_’s potential to restore both microbial and metabolic balance during sepsis.

Mechanistically, medical gases may reshape the microbiome indirectly by modifying the intestinal microenvironment [96]. H_2_ and H_2_S reduce epithelial oxidative stress and alter mucus and redox conditions that influence bacterial fitness, while CO and H_2_S can modulate perfusion and epithelial metabolism, thereby affecting nutrient availability for commensals versus pathobionts. These host-mediated shifts help create a luminal milieu that favors recovery of barrier-supporting taxa. H_2_S also exerts regulatory effects on microbial ecology. At low concentrations, H_2_S maintains mucus integrity, preserves the spatial structure of microbial biofilms, and prevents the emergence of invasive pathobionts, thereby contributing to immune homeostasis and inflammation resolution [97].

In contrast, the effects of CO on the gut microbiome under therapeutic conditions have not been well studied. However, exposure to elevated levels of CO, such as those observed in poisoning cases, has been associated with impaired barrier function and altered microbial composition. Both clinical and experimental studies have reported increased abundance of Desulfovibrio and Streptococcus, along with metabolic disturbances including insulin resistance and abnormal lipid metabolism, linking CO-related dysbiosis to systemic disease risk [98].

Recent studies have begun to examine how medical gases influence the microbiome in sepsis. In a murine model of sepsis-associated encephalopathy, H_2_ therapy partially restored dysbiosis, increasing *Verrucomicrobia* and *Akkermansia* while reducing *Lactococcus*, *Lactobacillus*, and *Escherichia-Shigella*. H_2_ treatment also altered serum and brain metabolite profiles, enriching neuroprotective metabolites and modifying amino-acid and energy pathways, suggesting that H_2_ may restructure microbial and metabolic networks during sepsis [95].

Microbiota-derived H_2_S shows a dualistic role: low levels support mucus integrity and biofilm organization, whereas excessive production by sulphate-reducing bacteria disrupts the gut barrier and promotes dysbiosis [99]. However, no studies have directly evaluated exogenous H_2_S donors on the sepsis-associated microbiome, and most evidence comes from non-sepsis disease models. Data on CO are even more limited. Observations of CO-related dysbiosis largely stem from poisoning cases; whether therapeutic low-dose CO or CORMs affect the microbiome in sepsis remains unknown.

Overall, while H_2_ and H_2_S appear capable of modulating the gut microbiome and metabolome, robust evidence in sepsis is lacking. Future work should incorporate metagenomic and metabolomic approaches to determine whether medical gases meaningfully alter microbial diversity and function in human sepsis.

While these findings suggest that medical gases may influence the gut microbiome, most available evidence remains associative or indirect, and is largely derived from preclinical or non-sepsis models. Accordingly, microbiome-related effects of medical gases should be interpreted cautiously until direct mechanistic and human data in sepsis become available.

## 7. Medical Gases and Autophagy

Autophagy, a cellular degradation process essential for maintaining homeostasis, plays a dual role in sepsis by regulating inflammation and cellular stress responses. While moderate autophagy confers cytoprotection by removing damaged organelles and controlling inflammation, excessive or dysregulated autophagy may contribute to tissue injury [100,101]. Emerging evidence indicates that several medical gases modulate autophagy to exert protective effects in sepsis, including within the gastrointestinal tract.

H_2_ therapy has been shown to mitigate sepsis-induced organ injury by activating autophagy and reducing endoplasmic reticulum (ER) stress [102]. Previous study demonstrates that H_2_-rich saline not only increases autophagy markers but also promotes mitophagy, thereby suppressing NLRP3 inflammasome activation and pyroptosis; inhibition of autophagy abolishes these protective effects [81]. In a murine model of sepsis-induced cardiomyopathy, H_2_ inhalation increased autophagy flux and mitophagy, and blocking autophagy with bafilomycin A1 reduced survival and reversed the cardioprotective effect of H_2_ [103]. In a murine CLP model, H_2_-rich saline increased autophagy markers (microtubule-associated protein 1 light chain 3-II, Beclin1) and downregulated ER stress proteins, resulting in decreased inflammation and improved survival [104]. Although gut-specific effects were not independently detailed, these pathways are likely relevant to intestinal protection.

H_2_S also regulates autophagy in the gut through key signaling pathways, including phosphoinositide 3-kinase/protein kinase B/mammalian target of rapamycin (mTOR), 5′ adenosine monophosphate-activated protein kinase (AMPK), and JNK [105]. In intestinal tissue, exogenous H_2_S donors such as NaHS or GYY4137 restore autophagic flux, reduce epithelial apoptosis, and maintain barrier integrity. H_2_S additionally promotes mitophagy and suppresses NLRP3 inflammasome activation, reinforcing mucosal protection during sepsis. In a septic cardiomyopathy model, NaHS treatment decreased autophagy-related proteins and improved myocardial function, suggesting that H_2_S attenuates cellular injury by inhibiting excessive autophagy via the AMPK/mTOR pathway [102].

CO demonstrates gut-specific autophagy-mediated protection. In a CLP model of sepsis, CO therapy increased survival through induction of Beclin-1-dependent autophagy and enhanced phagocytic clearance of bacteria; reductions in bacterial burden and inflammatory cytokines were observed in wild-type but not Beclin-1 heterozygous mice, highlighting the requirement of autophagy for CO’s protective effects [77]. In a murine sepsis model, CO-conditioned mesenchymal stromal cells (CO-hMSCs) reduced neutrophil infiltration and epithelial cell death in the ileum. These effects were abolished by autophagy inhibition, confirming the role of CO-induced autophagy. CO-hMSCs also attenuated local inflammation via extracellular vesicle signaling, highlighting autophagy as a critical mechanism in maintaining ileal immune and epithelial homeostasis during sepsis [106].

## 8. Comparative Analysis of Cross-Talk and Synergy Among Medical Gases

While H_2_, CO and H_2_S each confer gut-protective effects through unique mechanisms, emerging evidence suggests these gasotransmitters do not act in isolation. Reciprocal regulation of biosynthesis and signaling may create opportunities for complementary or even synergistic therapies. For instance, CO binds to the heme domain of CBS, the enzyme responsible for endogenous H_2_S synthesis, thereby switching the transsulfuration pathway toward remethylation and suppressing H_2_S production [107]. Conversely, H_2_S donors activate the Nrf2-HO-1 pathway, increasing heme oxygenase-1 expression and endogenous CO generation. Such bidirectional cross-talk has been implicated in the regulation of bile secretion and mesenteric microcirculation: CO attenuates H_2_S levels and stimulates bicarbonate-rich bile flow, whereas genetic or pharmacologic inhibition of H_2_S synthesis blunts this response. These findings underscore that CO and H_2_S can modulate each other’s bioavailability and physiological actions in the digestive system.

Synergistic interactions between H_2_ and the HO-1/CO axis have also been reported outside sepsis models. In mice with paclitaxel-induced neuropathy, combined treatment with cobalt protoporphyrin IX (a HO-1 inducer) and H_2_-rich water produced greater suppression of NLRP3 inflammasome activation, lipid peroxidation and inflammatory mediators than either treatment alone; the combination markedly up-regulated NRF2, HO-1 and downstream antioxidant enzymes [108]. Although similar combination studies are lacking in sepsis, these data suggest that co-administration of different medical gases or gas donors might allow lower doses and amplify gut-protective effects by simultaneously targeting multiple pathways (e.g., Nrf2 activation, autophagy/mitophagy, vasoregulation and immune modulation). Given that all three gases converge on TJ stabilization and NF-κB inhibition through distinct upstream mechanisms, exploring multi-gas strategies could yield additive benefits while mitigating the toxicity associated with high-dose single-gas therapy. Future research should examine optimal dosing, timing and sequence of combined gas therapies in sepsis models to fully harness their potential synergy.

**Figure 3:**
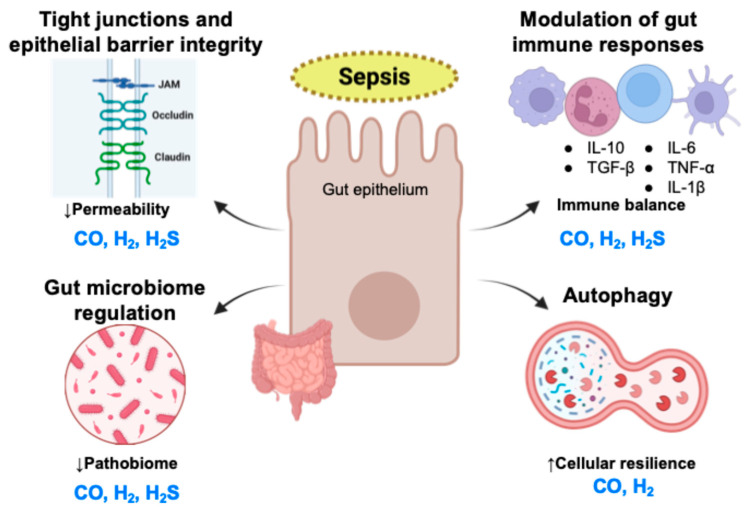
Integrated schematic of gut-protective mechanisms of medical gases during sepsis.

A consolidated schematic summarizing the multifaceted gut-protective actions of medical gases is presented in Figure 3.

This figure summarizes the major pathways through which hydrogen (H_2_), carbon monoxide (CO), and hydrogen sulfide (H_2_S) help preserve gut integrity in sepsis. Medical gases stabilize tight-junction structure to reduce epithelial permeability, modulate excessive inflammatory responses, and support microbial balance by limiting dysbiosis. In addition, H_2_ and CO enhance autophagy, promoting mitochondrial quality control and cellular resilience. Together, these coordinated actions mitigate septic gut injury and help maintain epithelial homeostasis.

IL, interleukin; TNF, tumor necrosis factor; TGF, transforming growth factor.

## 9. Comparison with Existing Gut-Protective Strategies

Several non-gas interventions aim to preserve gut integrity in sepsis. Probiotics and synbiotics restore microbial diversity and enhance short-chain fatty acid production, thereby strengthening TJs and modulating immunity. Enteral nutrition rich in fiber and immunonutrients (e.g., arginine, glutamine) supports barrier function and immune responses [109]. Immunomodulatory agents such as glucagon-like peptide-2 analogues, epidermal growth factor and anti-cytokine therapies are being tested to enhance mucosal repair [110]. Medical gases may complement these strategies by targeting oxidative stress and inflammatory pathways not addressed by microbiome-based interventions. For instance, combining H_2_ therapy with probiotics could simultaneously reduce ROS and replenish beneficial bacteria, while CO or H_2_S donors could synergize with nutritional support to enhance mucosal blood flow and healing. Head-to-head and combination studies are needed to assess additive benefits and to tailor interventions to patient endotypes.

## 10. Perspectives and Future Directions

Sepsis is a heterogeneous syndrome composed of distinct clinical phenotypes and biological endotypes, with important implications for gas-based therapies [111]. Gut-targeted medical gases may be most effective in subphenotypes characterized by early intestinal barrier disruption, oxidative stress, or hyperinflammatory responses. In this context, biomarker-guided stratification—such as markers of intestinal permeability, inflammation, or redox imbalance—could help identify patients most likely to benefit. Accordingly, these considerations support phenotype-driven trial designs that align patient selection and timing of intervention with dominant pathophysiology, rather than a uniform “one-size-fits-all” approach.

Within this precision-medicine framework, medical gases represent a promising therapeutic strategy in sepsis by preserving gut integrity, a key driver of systemic inflammation and multi-organ dysfunction. Among them, H_2_, CO, and H_2_S exert gut-targeted effects, including stabilization of tight junctions, modulation of immune responses, and interactions with the gut microbiome. These effects arise from their rapid diffusion into the intestinal mucosa and engagement of redox- and inflammation-related signaling pathways. Mechanistically, H_2_ activates the Nrf2/HO-1 pathway, CO stabilizes junctional integrity via NF-κB and MLCK modulation, and H_2_S enhances barrier defenses through protein persulfidation and Nrf2 activation, collectively suppressing pro-inflammatory signaling [112]. In addition, all three gases reprogram local immune populations and interact with the gut microbiota, further supporting their organ-selective protective actions. Although most evidence originates from preclinical models, selected translational observations reinforce the clinical potential of medical gases. Notably, exogenous H_2_S administration (via NaHS) significantly ameliorated NSAID-induced gastric injury in rats by preserving mucosal blood flow, reducing leukocyte adhesion, and attenuating cytokine upregulation [96,113]. These mechanisms are analogous to those underlying gut injury observed in sepsis. While the preclinical evidence is robust, translational challenges remain, including dose optimization, delivery systems, and safety validation, particularly for CO and H_2_S, which have narrow therapeutic windows [114,115].

Translating these findings into clinical practice faces several challenges, including dose extrapolation from animal models and the narrow therapeutic windows of CO and H_2_S. Safe application requires precise, low-dose delivery systems and careful patient selection, as factors such as lung function, gut permeability, and microbiome composition may influence gas uptake. Importantly, available human data indicate that low-concentration H_2_ inhalation is well tolerated, and CO/H_2_S are administered at doses far below those affecting daily activity [73]. These considerations are essential for designing early clinical trials in sepsis.

Beyond conventional inhalation-based delivery, emerging concepts such as enteral ventilation—including intrarectal oxygen administration and perfluorocarbon-based approaches—have been proposed as alternative strategies to improve tissue oxygenation while limiting systemic hyperoxia [116,117]. Early proof-of-concept studies suggest potential benefit under conditions of hypoperfusion. In clinical settings, medical gases may be administered during spontaneous breathing or controlled mechanical ventilation, each with distinct trade-offs in dose control, safety, and feasibility. As such, careful consideration of administration route and dosing strategy will be essential for clinical translation.

Future studies should prioritize gut-specific outcomes and biomarker-based stratification to identify gas-responsive sepsis endotypes. Combination strategies with probiotics, nutritional support, or immunomodulators may enhance therapeutic efficacy. Well-designed clinical trials evaluating efficacy, safety, and pharmacodynamics are needed to translate these approaches into precision, organ-targeted sepsis care.

## 11. Conclusions

Medical gases represent a novel class of therapeutics with multifaceted roles in preserving gut integrity during sepsis. By modulating oxidative stress, inflammation, immune responses, and microbiome balance, gases such as H_2_, CO, and H_2_S offer organ-targeted strategies to mitigate sepsis-induced intestinal dysfunction. While promising preclinical evidence supports their potential, further translational research and clinical trials are essential to validate their efficacy, optimize delivery methods, and ensure safety. Integrating medical gases into precision sepsis care may enable a shift from generalized immunosuppression to target enhancement of gut-associated protective responses.

## Figures and Tables

**Table 1:** Summary of mechanistic, preclinical, and clinical evidence for gut-targeted medical gases in sepsis.

Gas	Category	Key Gut-Protective Mechanisms	In Vitro/Cellular Evidence	Animal Sepsis Models (Species; Model)	Main Gut-Related Outcomes	Human Data	Translational Readiness	Key References
H_2_	Therapeutic medical gas (not gasotransmitter)	Selective antioxidant effects; Nrf2/HO-1 activation; suppression of NF-κB; autophagy/mitophagy induction; indirect microbiome modulation	Intestinal epithelial cells: reduced oxidative stress and apoptosis; Nrf2-dependent cytoprotection	Mouse/rat; CLP, endotoxemia, crush syndrome	Preserved TJ proteins (ZO-1, occludin); ↓ permeability; ↓ bacterial translocation; improved survival	Human safety data in non-sepsis conditions; no RCTs in sepsis	Preclinical strong; early clinical feasibility	[24,27,70,71,72,73]
CO	Core gasotransmitter (HO-1/CO axis)	HO-1-dependent signaling; inhibition of NF-κB and MLCK; Beclin-1-dependent autophagy; enhanced bacterial clearance	Epithelial & immune cells: TJ stabilization; reduced inflammatory signaling	Rat/mouse; CLP; CORM-2/3; HO-1 induction	Maintained TJ structure; ↓ MLC phosphorylation; ↓ inflammation; ↓ bacterial translocation	Phase I trial in sepsis-induced ARDS (feasible, well tolerated)	Early clinical translation (narrow therapeutic window)	[25,28,74,75,76,77]
H_2_S	Core gasotransmitter	Protein persulfidation (e.g., Keap1); Nrf2 activation; NF-κB and inflammasome suppression; MLCK-MLC inhibition	Epithelial and immune cells: enhanced antioxidant defenses; reduced cytokine release	Mouse/rat; CLP; NaHS, GYY4137, AP39	Preserved TJ integrity; ↓ cytokines; ↓ organ injury; improved survival (dose-dependent)	No direct sepsis trials; clinical donors (e.g., sodium thiosulfate) used in other indications	Preclinical strong; clinical indirect	[26,60,61,78]
CH_4_	Emerging candidate (context only)	Mitochondrial protection; anti-inflammatory effects; NF-κB suppression	Limited cellular data	Rodent/swine; advanced experimental sepsis	Reduced organ dysfunction; barrier protection reported	No human sepsis data	Exploratory/context	[63,64]
NO	Core gasotransmitter (context only)	Vascular and immune regulation; context-dependent effects	Extensive literature	Various models	Gut-specific protective effects inconsistent	Widely used clinically (non-gut targets)	Context only	[23]
N_2_O/N_2_	Classical medical gases	Anesthetic/ventilatory functions	—	—	No gut-protective signaling evidence	Established clinical use	Not gut-targeted	[65]

## Data Availability

No new data were created or analyzed in this study. Data sharing is not applicable to this article.

## Abbreviations

The following abbreviations are used in this manuscript:

AMPK	5′ adenosine monophosphate-activated protein kinase
CBS	Cystathionine-β-synthase cystathionine-γ-lyase (CSE)
CLP	Cecal ligation and puncture
CO	Carbon monoxide
CORM	Carbon monoxide-releasing molecule
CSE	Cystathionine-γ-lyase
ER	Endoplasmic reticulum
H_2_	Hydrogen
H_2_S	Hydrogen sulfide
HMGB1	High mobility group box 1
HO	Heme oxygenase
IKK	IκB kinase
IL	Interleukin
JNK	c-Jun N-terminal kinase
Keap1	Kelch-like ECH-associated protein 1
MAPK	Mitogen-activated protein kinase
MLC	Myosin light chain
MLCK	Myosin light chain kinase
MSC	Mesenchymal stromal cell
mTOR	Mammalian target of rapamycin
NF-κB	Nuclear factor Kappa B
NLRP	Nucleotide-binding domain, leucine-rich-containing family, pyrin domain-containing3
Nrf2	Nuclear factor erythroid 2-related factor 2
ROS	Reactive oxygen species
TCR	T-cell receptor
TGF	Transforming growth factor
TLR	Toll-like receptor
TJ	Tight junction
TNF	Tumor necrosis factor-alpha
TNFR	Tumor necrosis factor receptor
ZO	Zonula occludens
